# Evaluation of intestinal tuberculosis by multi-slice computed tomography enterography

**DOI:** 10.1186/s12879-015-1325-x

**Published:** 2015-12-22

**Authors:** Jing Zhao, Min-Yi Cui, Tao Chan, Ren Mao, Yanji Luo, Indira Barua, Minhu Chen, Zi-Ping Li, Shi-Ting Feng

**Affiliations:** Department of Radiology, The First Affiliated Hospital, Sun Yat-Sen University. 58th, The Second Zhongshan Road, Guangzhou, Guangdong 510080 China; Department of Radiology, Hospital of Stomatology, Guanghua School of Stomatology, Sun Yat-Sen University, Guangzhou, Guangdong China; Medical Imaging Department, Union Hospital, Hong Kong. 18 Fu Kin Street, Tai Wai, Shatin, NT Hong Kong; Department of Gastroenterology, The First Affiliated Hospital, Sun Yat-Sen University. 58th, The Second Zhongshan Road, Guangzhou, Guangdong 510080 China

**Keywords:** Tuberculosis, Intestine, Tomography, Spiral computed

## Abstract

**Background:**

Multi-slice computed tomography enterography (MSCTE) is now widely used to diagnose and monitor intestinal disease. Preliminary studies suggest that MSCTE may be useful in detecting intestinal tuberculosis (ITB). We sought to assess the use of MSCTE for the diagnosis of ITB in our medical center.

**Methods:**

In this retrospective study, 15 patients (11 males and 4 females, 6 to 65 years old) were enrolled and diagnosed with ITB by MSCTE. Diagnosis were confirmed by pathology or clinical criteria. Two experienced abdominal radiologists evaluated the images and defined the location, number, shape, edge, surrounding tissue alterations of ITB and other associated changes in the peritoneum, mesentery and solid abdominal organs.

**Results:**

The interval between the onset of symptoms and diagnosis varied from 20 days to 10 years. The most common symptom was abdominal pain (80 %). The ileocecum was the most common site affected by ITB (87 %). Morphological MSCTE findings were variable and included multi-segmental symmetric intestinal mural thickening found in 6 patients (40 %), solid masses found in 9 patients (60 %), and enlarged lymph nodes (LNs) found in 13 (87 %) patients. Non-enhancing central necrosis and rim enhancement were noted in 10 patients (67 %).

**Conclusions:**

Characteristic MSCTE findings of ITB include solid mass or multi-segmental symmetric mural thickening involving the ileocecal area and rim enhanced LNs. Knowledge of these features in combination with a high index of suspicion can be useful in early diagnosis of ITB.

## Background

Tuberculosis (TB) remains a major health problem particularly in developing countries [1]. According to the 2007 World Health Organization report, there are approximately 8.8 million new cases of TB each year with an annual mortality of over 1.6 million [2]. The intestinal tract is the sixth most common extra-pulmonary site of presentation [3]. Early diagnosis of intestinal tuberculosis (ITB) remains difficult due to its non-specific clinical presentations and its high resemblance to malignancy or other inflammatory diseases in radiology and colonoscopy [3–6]. A definitive diagnosis requires surgical biopsy. The early diagnosis of ITB is essential, as the disease is curable if treated early. In addition, the high morbidity and mortality associated with its complications, such as intestinal obstruction, perforation and hemorrhage, may therefore be avoided [7–9].

Small bowel barium enema, remains as a conventional and common method to evaluate intestinal diseases [10]. Some findings of ITB in small bowel barium enema has been reported, including mucosal thickening, spasticity and irregular contours, but extramural abnormality in the mesentery, omentum and lymph nodes (LN) cannot be identified [10]. Ultrasonography assessment is limited by intra-luminal bowel gas and obesity of the patients [11]. Regular multi-slice computed tomography (MSCT) and magnetic resonance imaging (MRI) can demonstrate alterations of the intestinal wall, mesentery, omentum and LNs with limited visualization of the intestinal mucosa and lumen. Multi-slice computed tomography enterography (MSCTE) or magnetic resonance enterography (MRE) evaluate both mural and extramural abnormalities and are promising tools for the evaluation of bowel diseases including Crohn’s disease (CD) and ITB [12, 13]. MRE can be technically challenging especially in patients with inadequate bowel distension where the image quality can be limited by gas artifacts. Few studies have been done conducted to evaluate the use of MSCTE in illustrating ITB manifestations [14, 15]. The purpose of this study was to evaluate the efficacy of MSCTE in diagnosing ITB.

## Methods

### General information

In accordance with ethical guidelines for human research, and compliant with the Health Insurance Portability and Accountability Act (HIPAA), this study was approved by the research ethical committee of The First Affiliated Hospital, Sun Yat-Sen University. Written informed consent was obtained from each adult patient or from the parents of the patients younger than 18 years old.

The diagnosis of ITB was established when at least one of the following criteria was met [7, 16]: 1) histological demonstration of caseating granulomas; 2) identification of acid-fast bacilli (AFB) in a histological specimen; 3) clinical, colonoscopic, radiological and/or surgical evidence of ITB associated with proven TB elsewhere and dramatic response to anti-tuberculous treatment. With regard to “TB elsewhere”, presence of active pulmonary tuberculosis (PTB) [17] and/or tuberculous peritonitis [18, 19] were considered positive.

According to the diagnostic standards above, 15 patients (11 male, four female; age of 6 ~ 65 years; mean age of 36 ± 18 years) with ITB were retrospectively included in our group from September 2006 to June 2012. Among these patients, 10 cases met the criteria for histopathological demonstration of granulomas with caseation, three cases met the criteria for identification of AFB in a histological specimen, and two met the criteria for colonoscopic, radiological and/or surgical evidence of ITB associated with proven TB elsewhere and response to anti-tuberculosis treatment without subsequent recurrence. At the same time, the patients’ history and family history of TB; purified protein derivative (PPD) skin test response; histopathology findings; AFB staining; TB culture reports, chest and abdominal imaging results; upper gastrointestinal endoscopy, colonoscopy, and laparoscopy results; and response to anti-tuberculosis treatment, surgical procedure, patient clinical course and complications were also recorded.

### Examination methods and instrumentation

All patients underwent cleansing enemas on the night before the examination and fasted for at least 6 h. To achieve adequate bowel distension, patients drank 2.5 % Mannitol 1 h (1.6-2.0 L), 45, 30, and 15 minutes (0.4-0.5 L, respectively) before CT scan. In addition, five patients were injected with normal saline or air through the anus to fully expand their rectum. CT was performed from the dome of the diaphragm to the level of ischial tuberosity with the patients in a supine position in a 64-detector-row scanner (Aquilion64, Toshiba Medical System, Tokyo, Japan). Tri-phasic images were acquired before and after (23–25 s and 50–60 s, respectively) intravenous administration of 50–100 mL of iodinated contrast medium (Ultravist 300, Schering, Berlin, Germany) at the rate of 3–4 mL/s. The acquisition parameters were 120 kV, 200–250 mAs, 64 × 0.5 mm collimation, 0.9 pitch, and 0.5 mm slice thickness.

### Image processing

All data were transferred to the workstation (Vitrea2, Toshiba Medical System, Tokyo, Japan) for multi-planar reformations (MPR), maximum intensity projections (MIP), curved planar reformation (CPR), and volume rendering technique (VRT). Two abdominal radiologists (ZP L and ST F) with 30 and 15 years of experience in abdominal imaging, respectively, reviewed the original and reconstructed images. The readers were aware of the diagnosis of ITB, but not the results of other clinical, endoscopic, and surgical findings. Two different window levels at 200/50 and 500/50 HU were used to display the intestinal wall and surrounding tissues. Location, number, shape and margins of intestinal lesions, involvement of surrounding fat, LN, other solid abdominal organs, peritoneum and mesentery were recorded. Intestinal wall measurements > 3 mm in thickness with distended lumen were considered highly suspicious of ITB lesions [20]. Reader discrepancies were resolved by consensus after re-evaluation of the images together.

## Results

The clinical data of the patients are summarized in Table 1.

**Table 1:** The clinical data of patients

	Patients (*n =* 15)
Sex (Male/Female)	11/4
Age (year)	
Range	6 ~ 65
Median	36
Disease duration	
Range	20 days ~ 10 years
Symptoms and abdominal physical symptoms	
Abdominal pain	12 (80 %)
Abdominal distension	3 (20 %)
Abdominal mass	1 (7 %)
Ascites	3 (20 %)
Fever	4 (27 %)
Marasmus	2 (13 %)
Diarrhea	3 (20 %)
Previous history	Renal transplantation for 1
Contact history of tuberculosis	none
Positive PPD	7 (47 %)
Chest X-ray	
Active tuberculosis	4 (27 %)
Old pulmonary tuberculosis	2 (13 %)
Pleural effusion	1 (7 %)

### Clinical features of ITB

The interval between onset of symptoms and diagnosis varied from 20 days to 10 years. Abdominal pain (80 %) was the most common symptom. The PPD skin test was positive in seven (48 %) patients. Four patients (27 %) suffered from active PTB, and two patients (13 %) had changes in PTB. One patient had right pleural effusion. Three patients had moderate ascites. Complications were found in nine patients, including incomplete (*n =* 6) or complete (*n =* 1) intestinal obstruction, intestinal perforation (*n =* 1), and intestinal hemorrhage (*n =* 1).

### Imaging features of ITB

#### Location

**Fig. 1:**
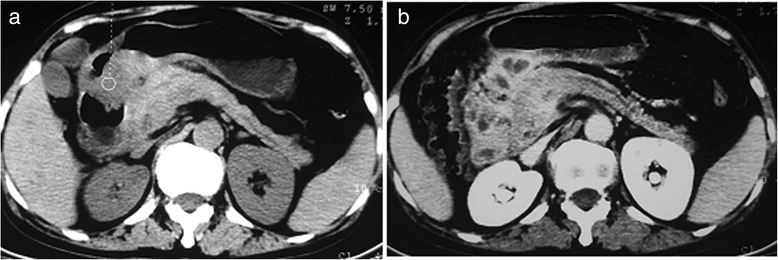
Duodenal tuberculosis. A patient had a history of abdominal pain for several months. **a**: heterogeneous solid mass located in duodenum. **b**: rim enhancement with non-enhancing center was shown after contrast

There were 13 patients (87 %) who had ITB involving the ileocecum, which was the most commonly affected site. One patient had a single short segmental involvement in the ileum, and one patient with ITB manifested with a solitary duodenal mass (Fig. 1).

### Number, shape, edge, surrounding tissue alterations

**Fig. 2:**
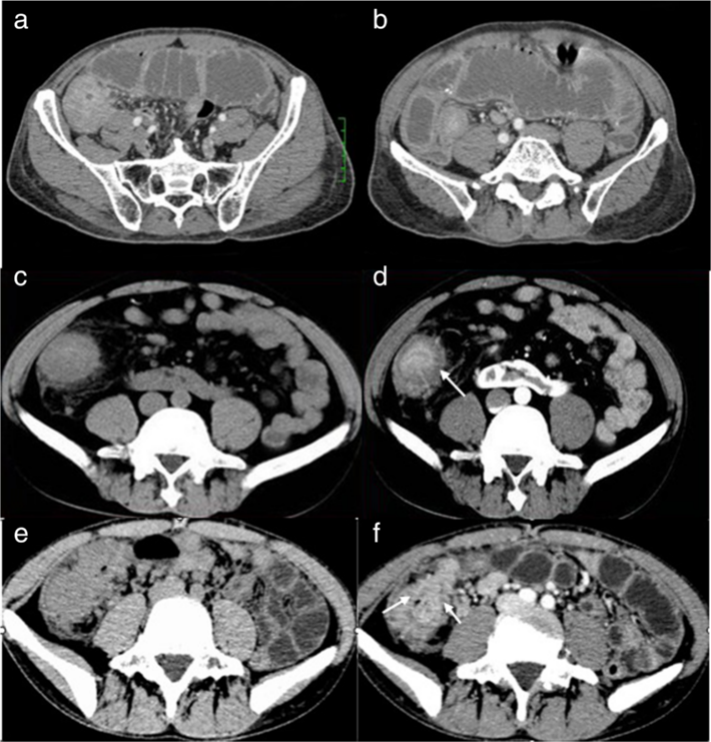
Different enhancement patterns of the solid masses. **a**,**b**: intestinal tuberculosis (ITB) in a old man with a history of left lower quadrant (LLQ) pain and abdominal distension for 20 days. A solid mass located in ileocecum that was misdignosed as colon carcinoma before surgery. The masses were homogeneously enhanced and the lumen of intestinal was narrow. **c**,**d**: ITB in a middle age man with a history of right lower quandrant (RLQ) pain for two year. A solid mass in cecum-ascending colon was displayed as “target sign”, (hyperenhancing inner and outer layer with hypoenhancing intervening layer) (white arrow). **e**,**f**: ITB in a young man with a history of RLQ pain for half a month. A solid mass in ileocecum was displayed as heterogeneous enhancement with caseous necrosis (white arrow)

Nine patients (60 %) mainly manifested with solid intestinal masses, including one patient who had a co-existing manifestation of multiple segments of symmetric intestinal mural thickening. Based on different enhancement of the solid masses, three types of imaging phenotypes have been identified, including the following: a, five solid masses showing homogeneous hyper-enhancement; b, two masses manifested with a target appearance (hyper-enhancing inner and outer layer with hypo-enhancing intervening layer); and c, two masses manifested with heterogeneous contrast enhancement due to necrosis (Fig. 2).

**Fig. 3:**
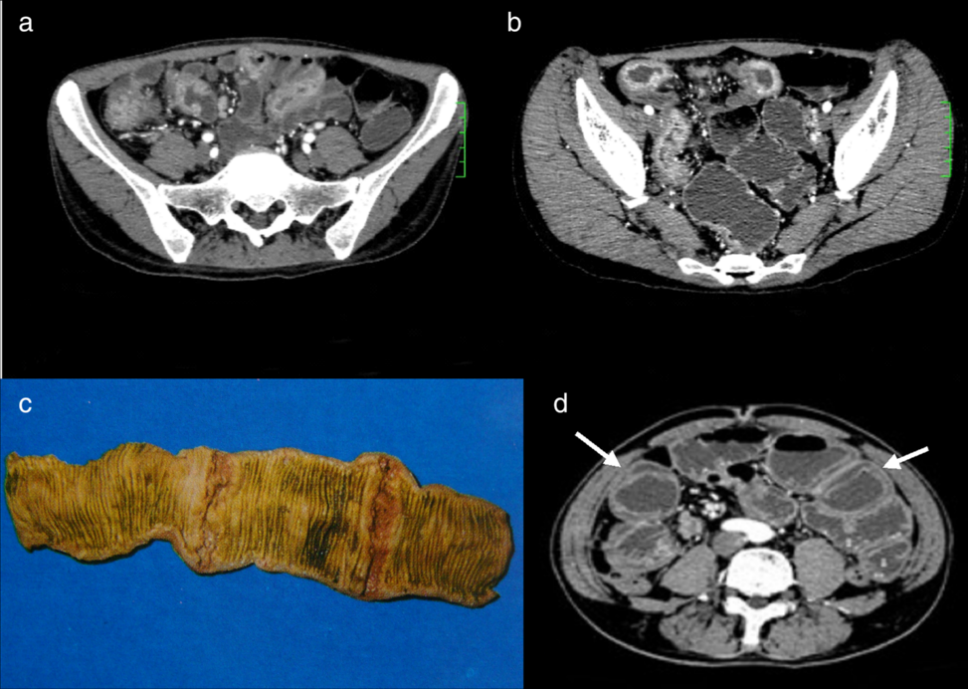
Symmetric intestinal mural thickening and double ring sign. Intestinal tuberculosis (ITB) in a old man with a history of left lower quadrant (LLQ) pain and abdominal distension for 20 days. **a**,**b**: Multi-slice computed tomography enterography (MSCTE) showed multiple segmental symmetric intestinal mural thickening with irregular ill-defined margins, and avidly enhancing intestinal mucosa. **c**: symmetric intestinal mural thickening confirmed as circumferential ulcer in the gross specimen. **d**: as a result of the distal small bowel obstruction, the intestinal wall was swollen and appeared as “double ring sign” (hyperenhancing intestinal mucosa and muscularis) after contrast enhancement (white arrow)

Single (*n =* 1) or multiple (*n =* 6) segments of symmetric intestinal mural thickening with irregular indistinct margins and luminal narrowing were observed in seven patients (47 %). Due to the distant intestinal obstruction, the edematous proximal bowel was depicted as a double ring sign (hyper-enhancing intestinal mucosa and muscularis) (Fig. 3).

### Adenopathy

**Fig. 4:**
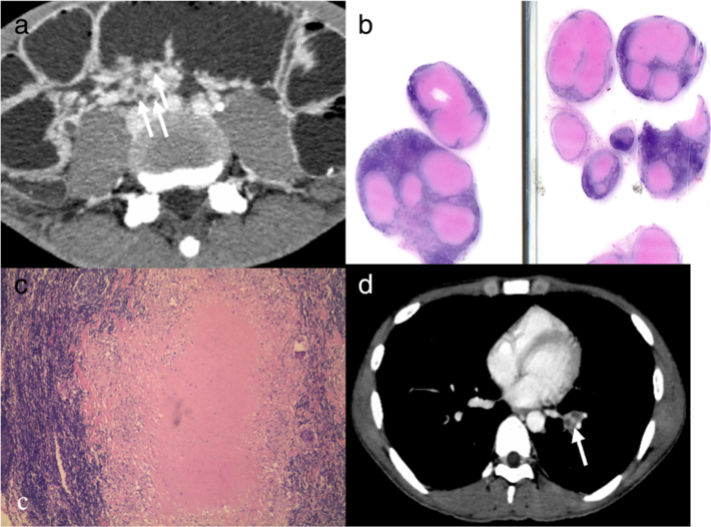
Peripheral rim enhancement of lymph nodes. Intestinal tuberculosis (ITB) in a young man had a history of right lower quandrant (RLQ) pain for three months. **a**: many lymph nodes were found in mesentery, typically manifested as peripheral rim enhancement (white arrow). **b**,**c**: histopathology and gross specimen confirmed peripheral rim enhancement based on caseous necrosis; **d**: multiple enlarged diaphragmatic lymph node with typical peripheral rim enhancement (white arrow)

Enlarged LNs were observed scattered throughout the mesentery tissue (the most common site), omentum, retroperitoneum or inguinal regions in 13 (87 %) patients. The size of the LNs varied widely, and the largest one was 4 cm across. LNs showing rim enhancement with non-enhancing central necrosis were noted in 10 (67 %) patients (Fig. 4). Calcified LNs were only detected in one patient.

### Others

Contrast-enhanced mesenteric arteries were observed as multiple tortuous tubules aligned on the mesenteric border of the ileum similar to the teeth of a comb in two (13 %) patients. High-density ascites was present in three patients with TB peritonitis. The complications of intestinal obstruction (*n =* 7) and perforation (*n =* 1) were all correctly diagnosed by MSCTE with 3-D reconstruction, but intestinal hemorrhage (*n =* 1) was not discernible on CT even in hindsight. Among the six patients undergoing chest CT, five had active PTB with rim-enhancing LNs found in two of them (Fig. 4).

## Discussion

Prior to CTE, using conventional CT for evaluations on the small intestine has been considered inadequate because pathology could easily be obscured amongst collapsed intestinal loops. With the rapid development of CT techniques, CTE has made it be possible. In CTE, the small intestine is expanded by oral ingestion of a neutral or negative contrast agent, such that mucosal and mural changes are clearly depicted [12]. One major challenge in performing good quality CTE is about the optimal volume and timing of oral contrast administration, balancing the quality of intestinal distension with patient tolerance and degree of side effects. Nowadays, CTE is considered as an effective imaging technique to detect and evaluate many gastrointestinal diseases [12, 13, 21]. Most CTE studies have concentrated on the diagnostic performance for the intestinal inflammation and intestinal complications of Crohn’s disease [22]. Reports on the utility of CTE in the diagnosis of ITB are still rare.

We retrospectively evaluated the clinical data and MSCTE images of 15 patients with ITB. We found that the ileocecum was the most common site affected by ITB. MSCTE showed that nine patients (60 %) mainly manifested with solid masses, and six patients (40 %) were shown as having multiple segments of symmetric intestinal mural thickening. Rim enhancement of LNs was found in 10 (67 %) patients, which also could be used to enhance the diagnostic confidence of ITB.

Prior studies also reported the ileocecum as the most commonly affected site of ITB [6, 11, 23, 24]. The abundance of lymphoid tissues, the relatively longer time for fecal stasis, the appropriate neutral pH environment and the absorptive transport mechanisms (increasing rate of fluid and electrolyte absorption) of the ileocecum work together to allow the swallowed mycobacterium to be absorbed and might contribute to the predilection of ITB [25]. The duodenum is the fourth most commonly affected site following the ileocecum, colon and jejunum, and greater than 90 % of duodenal TB was also found to have co-infections in other parts of the intestine [26]. However, in our study, one patient showed isolated duodenal TB.

Generally, there are three pathological types of ITB, as follows: ulcerative, hypertrophic and ulcerohypertrophic. The last two types display mass-like lesions and mimic malignancies. The bowel wall changes were variable, reflecting the different pathological types. Seven patients (47 %) in our study were found to have symmetrical, single or multi-segmental intestinal mural thickening due to ulcerations. Focal solid masses representing the hypertrophic or ulcerohypertrophic types were observed in nine patients (60 %).

ITB is also a chronic granulomatous disease with inflammatory and reparative phases. The individual lesions could be in different stages of inflammation and repair, such that the MSCTE findings vary between different lesions within the same patient. During the inflammatory phase, the length and size of lesions were larger with indistinct boundaries and were expected to enhance homogeneously. In our study, all of the lesions manifested with indistinct boundaries. Further, as inflammation could cause submucosal edema, the affected intestine wall or solid mass appeared as a ‘target sign’. On the contrary, during the reparative phase, the lesions tended to have more clearly defined boundaries with less contrast enhancement because of the fibrosis in the middle layer of the intestinal wall. In addition, fibrosis could further progress into stricture formation, resulting in abnormal peristalsis and mechanical obstruction. Proximal to the obstruction site, the bowel was often dilated with the edematous intestinal wall manifesting as submucosal edema and appearing as a double-ring sign on contrast-enhanced CT. In our study, seven patients with MSCTE showed these signs.

Lymphadenopathy was another common manifestation of ITB. The most common CT manifestation of diseased LN was rim enhancement with a low-density center, representing peripheral inflammatory reactions and central caseous necrosis. This imaging appearance was highly suggestive of ITB although other conditions such as metastasis, Whipple’s disease, lymphoma, and infection with mycobacterium avium-intracellulare can have a similar appearance [27]. In our research, LNs with rim enhancement were observed in 10 patients (67 %). In addition, the low-density necrotic components in affected LNs ranged from tiny dots to large areas involving the majority of an LN, which did not correlate with the size of the LN. The smallest necrotic node found in our study was only 6 mm in size. We also found that the artery phase of MSCTE was the best for detecting caseous necrosis. Moreover, we identified an interesting phenomenon in that two of our patients with the most severe TB symptoms and active PTB also showed signs of complete central caseous necrosis in their LNs. It is, therefore, surmised that activity of ITB may be reflected by the degree of necrosis in LNs, but this assumption requires further confirmation. On the other hand, only one patient in our study had calcified LNs, suggesting that calcified LNs might not be frequently observed.

Other changes associated with ITB found in our study included ascites (*n =* 3), mesenteric thickening and hypervascularity (comb sign; *n =* 2), suggesting that the comb sign is not a unique sign of Crohn’s disease as mentioned in previous literature [28]. Biochemically, the ascitic fluid showing high attenuation values (25–45 HU) indicated exudative fluid with high protein and cellular content. The scans showed that the peritoneal fluid was diffusely distributed throughout the peritoneal cavity, suggesting wet peritonitis as the possible cause [29].

ITB could accompany abdominal complications, including obstruction, perforation, and fistula formation. All of the complications in our study, with the exception of one with intestinal hemorrhage, were correctly diagnosed by MSCTE. Four of the patients (27 %) had imaging manifestation of PTB. Daucourt V et al. found that 69 % of extra-PTB presentations was associated with PTB [30]. In our study, the percentage was a little lower, and the reason may be that not all patients had thorax examination. Still, we recommend that a plain chest X-ray be obtained in clinically suspicious ITB patients because patients who have PTB are at a higher risk of secondary intestinal involvement.

Of the differential diagnoses of ITB, Crohn’s disease (CD) is at the top of the list. ITB and CD are both chronic granulomatous disorders with similarities in clinical presentation and pathology [31–34]. As discussed before, ileocecal region is the most common site of involvement in ITB [11, 23]. Isolated involvement of the ileocecal region is not found in CD, which typically involves the ileum whilst sparing of the ileocecal valve. The cecum may rarely be involved in direct contiguity with the ileum or colonic disease in ITB. Whilst the ascending colon may be involved in direct contiguity with the ileocecal region in ITB, colorectal involvement with small bowel disease is more often observed in CD than in ITB. Isolated colorectal involvement in ITB is rare compared with CD. Presence of hypodense LNs with peripheral rim enhancement in the mesentery and retro-peritoneum is rarely observed in CD, which also helps differentiate between the two. Furthermore, it is difficult to differentiate a solitary ITB from malignancy on imaging, especially in older patients [35]. Malignant stricture often occurs in elderly patients and manifests as solitary lesion, usually ranging from 2 to 6 cm long with irregular contours, intraluminal filling defects, and rigid, tapering margins. ITB is more commonly found in younger patients and often presents as multiple lesions [35]. Other differential diagnsises include Yersinia enterocolitis and Yersinia pseudotuberculosis, which preferentially affect the gut-associated lymphoid tissue and related LNs, respectively. These conditions commonly masquerade as acute appendicitis and ileitis or mesenteric adenitis in children and young adults [36].

One major limitation of this study is the relatively small case number, which is to a large extent due to the fact that ITB is a rare disease. Therefore, the imaging manifestations of ITB in CTE as reported in our study might not necessarily reflect the full spectrum of related imaging findings.

## Conclusions

ITB patients present with a variety of non-specific symptoms and signs. Chest X-rays should be conducted to look for PTB, which is often helpful in situations of diagnostic difficulty. MSCTE study is useful in the diagnosis of ITB, and the related changes in bowel, LNs, and complications can all be clearly observed in MSCTE. Among the different manifestations, ileocecal involvement and the presence of rim enhancing LNs may suggest the diagnosis of ITB.

## Abbreviations

AFB: acid-fast bacilli
CD: crohn’s disease
ITB: intestinal tuberculosis
LNs: lymph nodes
MRI: magnetic resonance imaging
MRE: magnetic resonance enterography
MSCTE: multimulti-slice computed tomography enterography
PPD: purified protein derivative
PTB: pulmonary tuberculosis
